# P2X7 Variants in Oncogenesis

**DOI:** 10.3390/cells10010189

**Published:** 2021-01-19

**Authors:** Anna Pegoraro, Elena De Marchi, Elena Adinolfi

**Affiliations:** Department of Medical Sciences, Section of Experimental Medicine, University of Ferrara, Via Luigi Borsari 46, 44121 Ferrara, Italy; anna.pegoraro@unife.it (A.P.); elena.demarchi@unife.it (E.D.M.)

**Keywords:** P2X7, P2X7 splice variants, P2X7 SNPs, cancer, leukemia, P2X7B

## Abstract

The P2X7 receptor for extracellular ATP is a well-established mediator of tumoral development and progression both in solid cancers and hematological malignancies. The human P2X7 gene is highly polymorphic, and several splice variants of the receptor have been identified in time. P2X7 single-nucleotide polymorphisms (SNPs) have been broadly analyzed by studies relating them to pathologies as different as infectious, inflammatory, nervous, and bone diseases, among which cancer is included. Moreover, in the last years, an increasing number of reports concentrated on P2X7 splice variants’ different roles and their implications in pathological conditions, including oncogenesis. Here, we give an overview of established and recent literature demonstrating a role for human P2X7 gene products in oncological conditions, mainly focusing on current data emerging on P2X7 isoform B and nfP2X7. We explored the role of these and other genetic variants of P2X7 in cancer insurgence, dissemination, and progression, as well as the effect of chemotherapy on isoforms expression. The described literature strongly suggests that P2X7 variants are potential new biomarkers and therapeutical targets in oncological conditions and that their study in carcinogenesis deserves to be further pursued.

## 1. Introduction

P2 purinergic receptors are plasma membrane proteins activated by nucleotides and subdivided into metabotropic P2Y and ionotropic P2X receptors. P2X receptors are ATP gated ion channels permeable to Na^+^, K^+^, and Ca^2+^ that assemble as homo or heterotrimers of seven different subunits (P2X1-7) [1]. The P2X7 subunit, with its 595 amino acids (aa), is the most extended protein in the P2X family, and it assembles to form a homo-trimeric receptor. Different domains form the P2X7 structure, including a short intracellular N-terminal tail (26 aa), a large extracellular loop (282 aa) containing the ATP binding site, two transmembrane helices (24 aa each), and a long cytoplasmic carboxy-terminal tail (239 aa) [2,3]. Among these domains, the C terminal tail is unique at P2X7, as it is not present in any other member of the P2X family, and it confers to the receptor the ability to interact with several intra-cytoplasmic and transmembrane proteins [4,5,6]. Moreover, the P2X7 C-tail is responsible for a peculiar activity triggered by the receptor: the opening of a big non-selective pore, which is known as the “macropore” [2,7,8]. The macropore is activated upon a long exposure of P2X7 to high concentrations of its ligand ATP, and it is permeable to aqueous solutes such as ethidium bromide, propidium iodide, YO-pro, and Lucifer Yellow [7]. The macropore formation is reversible, as the removal of ATP, in a few minutes from its addition, induces the reconstitution of cell membrane integrity [9]. Whether the macropore-related activities are dependent only on P2X7 permeabilization or also on accessory pathways or intracellular signaling is still an object of debate [10,11]. However, it is well accepted that the macropore’s opening is associated with cytotoxicity; instead, the tonic activation of P2X7 with limited ATP stimulation supports a trophic effect [12]. While the general tertiary structure of the P2X7 is comparable to that of other P2X receptors [13,14,15], that of the C terminal tail was missing until a recent study by McCarthy et al. [16]. The authors reported the presence of a C-cys anchor domain that, if palmitoylated, causes the typical non-desensitizing current associated with P2X7. Based on this molecular structure, a following study proposed that the P2X7 C terminal domain was acquired by genomic rearrangement from a P2X4-like gene in ancient jawed vertebrates generating the actual mammalian P2X7 [17]. Therefore, it is not completely surprising that some P2X7 splice variants, which function as a channel but not as macropore have been conserved through evolution [18]. Many cell types and tissues express the P2X7 receptor, but its role was mainly studied in the immune system, where its activity is associated with inflammatory conditions [19,20]. Indeed, P2X7-dependent activation of the NLRP3 inflammasome and caspase 1 and the consequent maturation of pro-IL1β is an established notion [21]. The P2X7 receptor is also central to the cytokine’s vesicular release from dendritic cells, macrophages, and microglia [22,23,24]. The implications of P2X7 activity in inflammatory and immune disorders, neuronal, cardiovascular, and metabolic diseases, and cancer have been recently appraised in several reviews [14,25,26,27,28,29,30,31,32]. Therefore, they are out of this manuscript’s scope. Here, we wish to give an overview on established and recent literature reporting the involvement of human splice and polymorphic variants of the P2X7 receptor in oncological conditions.

## 2. Human P2X7 Splice Variants and Single-Nucleotide Polymorphisms

**Figure 1:**
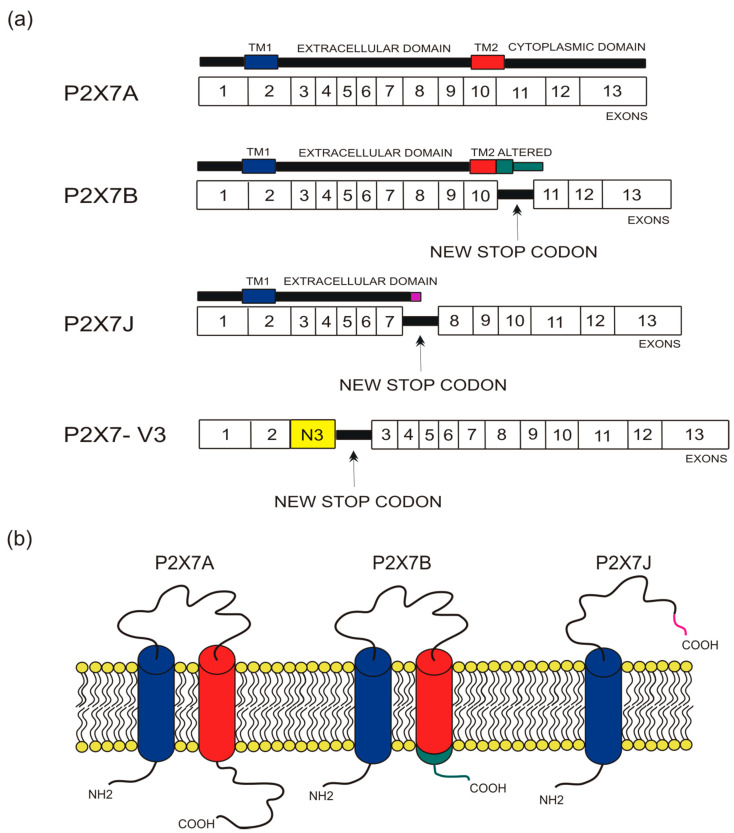
Depiction of human P2X7 receptor splicing variants described in this overview as involved in carcinogenesis and for which a published sequence is available. Different colors identify each region: transmembrane domain 1 (TM1) is in blue, transmembrane domain 2 (TM2) is in red, altered carboxy-terminal tails of P2X7B and P2X7J are respectively in green and in purple. (**a**) Schematic representation of the human P2X7 gene and its splice variants. The boxes represent the 13 exons, while the black lines between exons represent new introns. The full-length P2X7A variant derives from the original sequence formed by 13 exons. The P2X7B isoform is a truncated variant as it retains an intron between exons 10 and 11, which includes a new stop codon altering the carboxy-terminal tail. P2X7J is a truncated isoform that lacks exon 8 and carries a modified carboxy-terminal tail. P2X7-V3 gains an extra exon called N3 (yellow) and a stop codon; it does not give rise to a protein but instead acts as a short non-coding RNA. (**b**) Schematic representation of the full-length P2X7A and the truncated isoforms P2X7B and P2X7J expressed on the cell membrane.

**Figure 2:**
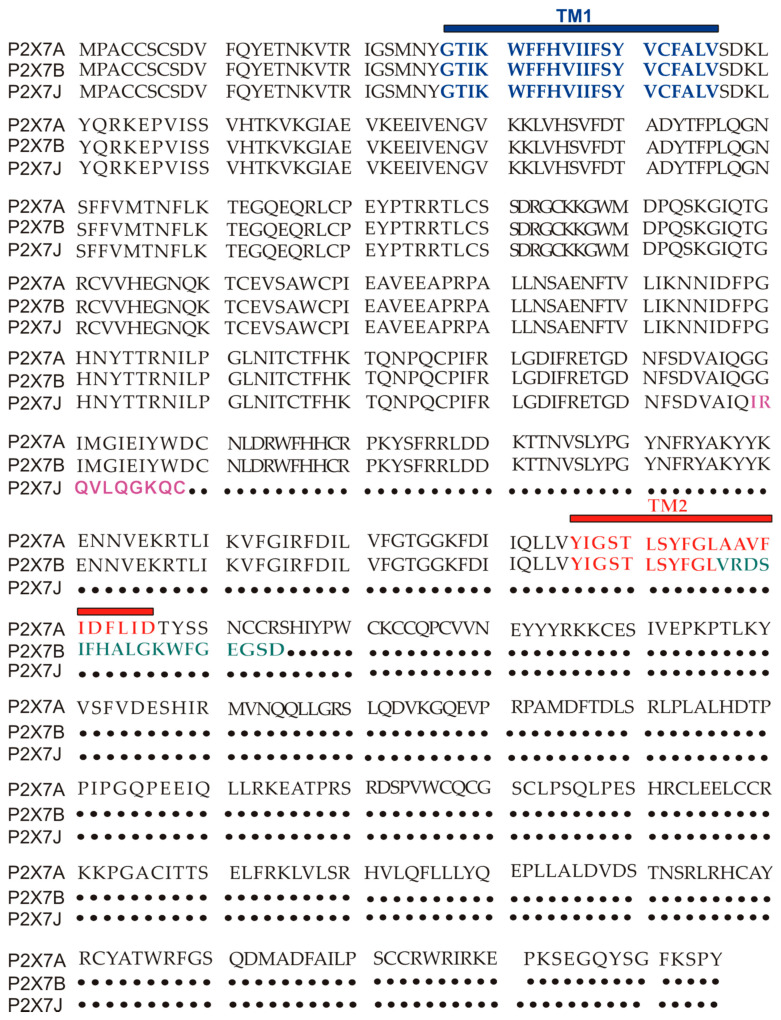
P2X7A (full length) and P2X7B and J amino acids sequence alignment. TM1 is in blue, while TM2 is in red. The ten aminoacids unique to the P2X7J isoform are in purple, while the 18 extra amino acids characterizing P2X7B and located after TM2 are in green.

The human P2X7 receptor gene is located on chromosome 12 and consists of 13 exons [33]; it is highly polymorphic and gives rise to several splice variants [34]. The splice variants, which were initially identified around 2005, are listed as (P2X7A-J) [18,35], where P2X7A is the mRNA coding for the complete protein and generally referred to as a full-length P2X7 receptor [36]. Among the other alternative mRNAs, P2X7B, P2X7E, P2X7G, and P2X7J give rise to truncated proteins that lack the extended C-terminal tail [18,35]. The splicing isoforms are generated either via the inclusion of a new exon or the exclusion of one or more genetic regions. Indeed, P2X7G and P2X7H have an inserted additional exon, while P2X7C, P2X7D, P2X7E, and P2X7F lack respectively exon 4, exon 5, exons 7 and 8, exons 4 and 8 [18,36]. Instead, P2X7I derives from a point mutation in the first intron of P2X7, leading to an extended truncation, and P2X7J is truncated after exon 7, making it non-functional (Figure 1 and Figure 2). Nevertheless, it acts as a dominant-negative on P2X7A [35]. On the contrary, P2X7B exerts a dominant positive activity at the main isoform [8,37]. The P2X7B variant was probably the most studied, as it is the only one to be functional as a small ion channel [8,18,37,38,39,40,41,42]. The P2X7B isoform retains an intron between exons 10 and 11, causing the addition of 18 extra amino acids after residue 346, which is followed by a stop codon [18] (Figure 1). Nevertheless, P2X7B EC50 is relatively high, and depending upon the cell type and experimental conditions tested, it is not always expressed and functional at the plasma membrane [8,37,41]. Another recently identified alternative mRNA for the human P2X7 is P2X7-V3 (Figure 1). This splice variant does not give rise to a functional protein but to a long non-coding RNA endowed with a protumoral activity [43]. Another conformational form of the receptor losing P2X7-dependent macropore formation is nfP2X7. In this case, currently, there is no reported sequence allowing us to define whether it is a splice variant or a polymorphic form of the receptor. However, we know that nfP2X7 is recognized by polyclonal antibodies raised against a peptide of human P2X7, including amino acids 200–216. This sequence is accessible as an antigen only in nfP2X7 and not on the wild-type receptor [44]. siRNA experiments have demonstrated that nfP2X7 is a product of the endogenous human P2X7 gene, thus strongly suggesting that this conformational variant could be originating from either alternative splicing or point mutations [45]. nfP2X7 is generally present as an intra-cytosolic protein, which can be translocated at the plasma membrane when the cells are exposed to high ATP concentrations, such as those present in the tumor microenvironment [45]. Similar to P2X7B, nfP2X7 is active as a small ion channel but not as an ethidium permeable macropore; however, unlike P2X7B, it does not exert a dominant positive effect on P2X7A [45].

**Figure 3:**
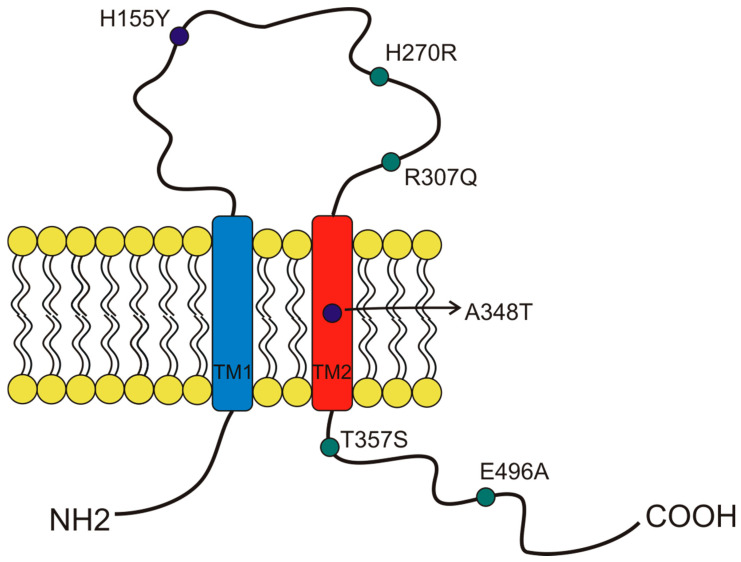
Schematic representation of the position of P2X7 receptor single-nucleotide polymorphisms (SNP) studied in cancer and covered by this overview. The gain of function SNPs are in blue, while the loss of function SNPs are in green.

More than 13,000 single-nucleotide polymorphisms (SNPs) of the P2X7 receptor were identified in the human gene. Among those SNPs, 10 were demonstrated to give rise to loss-of-function variants, and three were demonstrated to give rise to gain-of-function variants [34,36,46]. This significant variability was possibly due to the receptor’s central role in response to infection and other inflammatory conditions [19,20]. Several studies associated P2X7 SNPs with the predisposition to arise different diseases, influence cell functionality, and induce cancer development [34]. Here, we describe the characteristics of the SNPs that, to our knowledge, were so far associated with oncological conditions (Figure 3). The first human P2X7 SNP to be identified was the loss of function 1513A > C (rs3751143) variant characterized by the substitution of glutamic acid with alanine in position 496 (E496A) in the long C terminal tail of the receptor [47] (Figure 3). Experiments conducted on leukocytes homozygotes for this SNP revealed a nearly complete loss of activity both as a channel and macropore [47]. The effects of this SNP expression have been extensively studied in different cell types and were associated with several cell dysfunctions that involve the release of pro-inflammatory cytokines, cell death, and membrane molecules exposure [48]. These impairments were associated with susceptibility to tuberculosis infection in different populations [48], a high risk of sporadic Parkinson’s development in a Chinese population [49], and with the increase of bone fracture events in postmenopausal women [50]. As P2X7 1513A > C variant, also, the 946G > A (rs28360457) polymorphism causing the substitution of the arginine at amino acid position 307 with glutamine (R307Q) leads to a loss of function by changing the site of ATP interaction in the extracellular domain [51] (Figure 3). Similarly, the 835G > A (rs 7958311) SNP causes the substitution of histidine 270, which is located in the extracellular domain of P2X7, with an arginine (H270R), and the 1096G > C variant (rs2230911) leads to a serine to threonine change in position 357 of the carboxy-terminal tail (T357S) and both mutations cause P2X7 loss of function [52] (Figure 3).

Among the three gain-of-function polymorphisms known, the most recurrent P2X7 SNP in humans is the 489 C > T (rs208294), which carries a tyrosine instead of a histidine at position 155 in the extracellular domain (H155Y) [53] (Figure 2). This SNP increases the receptor function as both an ion channel and macropore and, interestingly, it corresponds to the wild-type allele in the mouse and rat P2X7, which is long known to have lower EC50 for agonists than the human isoform [2,33,54]. Hu and colleagues demonstrated that this polymorphism is associated with the activation of NLRP3 inflammasome and causes a rise of IL-1β and IL-18 secretion [55]. The notion that pro-inflammatory molecules’ secretion is one of the causes that can predispose to mood disorders and inflammatory diseases is an established one. Indeed, human P2X7 489 C > T SNP was associated with pathological conditions such as Alzheimer’s [56], Systemic Lupus erythematosus [55], HHV-6A infection, and related infertility [57], as well as cancer pain [58]. Moreover, it was co-associated with another gain-of-function SNP, the 1068G > A (rs17118119), with increased pain sensation in females affected by diabetic peripheral neuropathic pain [59]. The 1068G > A SNP gives rise to a protein characterized by the presence of threonine instead of alanine at amino acid position 348 (A348T), which is located at the second transmembrane domain. Monocytes carrying the 1068G > A SNP secreted 3-fold more IL-1β than those collected from P2X7 wt subjects when treated with LPS. Threonine 348 also increased P2X7-mediated ethidium uptake and Rb(+) efflux [60]. Based on these data, Stokes and colleagues proposed that this SNP could be a predisposing factor for the development of inflammatory, infectious, and psychiatric disorders [60]. The third gain-of-function variant discovered is the 523C > G, in which the arginine in position 166 of the extracellular domain is substituted by glycine (A166G) [61].

### 2.1. Role of ATP and P2X7 Receptor in Oncology

The P2X7 receptor and its ligand ATP recently emerged as important players in regulating solid cancer and leukemic growth as well as immune system/cancer cross-talk [30]. Indeed, ATP acts as a danger signal, meaning that it is a molecule abundant inside the cytosol and virtually absent in the extracellular space, and it is released upon necrotic cell death. Extracellular ATP activates an immune response interacting with P2X7 [19]. Accordingly, increased levels of extracellular ATP can be measured in the tumor microenvironment and are influenced by the principal anticancer treatments, including chemo and radiotherapy [62,63,64,65]. In a recent study, Kamata-Sakurai and colleagues demonstrated that extracellular ATP could be exploited to activate anti-tumoral therapeutics such as an anti-CD137 antibody only in the tumor milieu, thus increasing their tumor-suppressing activity while reducing unwanted systemic side effects [66]. ATP also modulates tumor-associated immune response via the direct activation of inflammatory pathways [67] or causing immune suppression via the generation of its degradation product adenosine through CD39 and CD73 ectonucleotidase activation [68]. P2X7 pharmacological blockade enhances tumor infiltration by T cells and diminishes the expression of CD39 and CD73, thus reducing immunosuppression in the tumor microenvironment [69]. Interestingly, expression of the P2X7 receptor itself is crucial to guarantee high extracellular ATP levels in the tumor bed. Indeed, P2X7-null mice show a reduced ATP concentration when bearing an experimental melanoma or leukemia [69]. As per tumor-promoting activities, P2X7 enhances cell proliferation [12,70,71], angiogenesis [70,72,73], matrix degradation [74], and metastatic spreading [38,74,75,76,77] in several neoplastic conditions. Accordingly, the efficacy of P2X7 blockers in reducing cancer growth and dissemination was demonstrated in several preclinical models of melanoma [69,78,79], neuroblastoma [72], breast [74,76,80], prostate [77], and pancreatic carcinoma [81], mesothelioma [82], and leukemia [42,69]. Interestingly, an antibody directed against nfP2X7 also proved efficacious in reducing basal cell carcinoma growth in a phase I clinical trial on humans [83]. These data are of sure relevance to develop new P2X7-targeting therapies, also considering that an increasing number of studies report an association between P2X7 overexpression and bad prognosis or response to therapy in oncologic patients [42,65,72,84,85,86,87]. The majority of these studies do not take into account known P2X7 variants, but some interesting progress was made in this field of research in last years that we cover in the present overview.

### 2.2. P2X7 Splice and Conformational Variants in Oncology

As mentioned above, one of the most studied splice variants of P2X7 is certainly P2X7B, whose role in cancer was investigated at first due to its transforming activity [8]. Indeed, in vitro evidence points to a protumoral role for the isoform, as P2X7B retains P2X7A growth-promoting activity while losing macropore-related cytotoxicity [8]. P2X7B was shown to activate the NFATc1 proliferative pathway, promote soft agar invasion, and ATP secretion [8,37]. The first study reporting a role for P2X7B in a solid cancer model was performed by our group and showed the expression of P2X7B in human osteosarcoma [37] (Table 1). We demonstrated by immunohistochemistry of osteosarcoma tissues that both P2X7 isoforms are expressed in these specimens with a prevalence for P2X7B. Experiments conducted on Te85 osteosarcoma cells transfected with P2X7A and P2X7B, either separately or together, showed the increased proliferation of all Te85 P2X7 clones in serum-starving conditions compared to control cells. Interestingly, when expressed in Te85, P2X7B strongly reduced bone deposition compared to untransfected controls, while P2X7A did not affect mineralization, thus suggesting a different role for the isoforms in osteosarcoma [37]. These data were further confirmed by a subsequent study on mesenchymal stem cells. Carluccio and colleagues reported a higher expression of P2X7B in stem cells acting as osteoblast precursors than in finally differentiated osteoblasts [39], suggesting that P2X7B could be promoting osteosarcoma formation by favoring an undifferentiated cell state. In a different study, Ulrich and colleagues investigated the role of bradykinin, a pro-metastatic factor, as a promoter of neuroblastoma metastasis in bone marrow, exploring different molecular pathways involved in cancer invasiveness, among which the P2X7 receptor is included [38]. In particular, they showed that the treatment of two different human neuroblastoma cell lines (CHP-100, SH-SY5Y) with bradykinin preferentially upregulated P2X7B as compared to P2X7A. Moreover, they observed that bradykinin treatment induces cell proliferation. All these data suggest that kinin could turn extracellular ATP, which is present in the bone marrow of neuroblastoma bearing-mice, into a growth-metastatic stimulus by causing the overexpression of P2X7B [38] (Table 1). Ziberi and colleagues centered another study examining P2X7 receptor splice variants in human glioblastoma stem cells (GSCs) [40]. The authors reported the expression of both P2X7 isoforms in GSCs examined from three different patients. Moreover, P2X7A and P2X7B levels were increased in GSCs cells following treatment with the ATP analog and P2X7 agonist 2′ [3′]-O-[4-benzoylbenzoyl]-ATP (BzATP). Pre-incubation of the cells with the P2X7 antagonist A438079 neutralizes the effect of BzATP on P2X7A and P2X7B expression. These data suggest the presence of a P2X7-activated autocrine loop that is able to upregulate the expression of both P2X7 isoforms. Finally, the authors suggest that the positive modulation of P2X7A and P2X7B following BzATP treatment might support GSCs invasion, and consequently, P2X7 could be a potential pharmacological target to treat glioblastoma [40] (Table 1). In 2020, in a study by Benzaquen and colleagues, the P2X7 variants were investigated in lung adenocarcinoma and tumor immune cells [41]. Interestingly, even though the expression of different P2X7 splice variants was observed in both cancer and immune infiltrate, the authors reported an upregulation of P2X7B, particularly in tumor immune cells. They also demonstrated that the expression of P2X7B correlates with the nature of immune infiltration within the tumor. In particular, they found a negative correlation between P2X7B expression and T and B lymphocytes while, on the contrary, there was an increase of myeloid cell content in patients expressing the higher levels of P2X7B [41] (Table 1). Unfortunately, nothing is known yet about P2X7B role in inflammatory responses classically attributed to P2X7. New findings relative to this last issue, such as demonstrating that P2X7B can activate the release of pro-inflammatory cytokines, will be of sure interest for purinergic and oncological scholars. Finally, in a recent study, we analyzed the differential expression of both P2X7 isoforms A and B in acute myeloid leukemia (AML) and the effect of P2X7 antagonism on their expression [42] (Table 1). We first investigated the expression of the two isoforms in a cohort of patients affected by AML or by myelodysplastic syndrome (MDS), a pre-cancerous blood disease that is often leading to AML development. AML patients were further subdivided according to the diagnostic phases and response to treatment in newly diagnosed untreated subjects (*de novo*), relapsing patients with a pathology return after chemotherapy, and remitting patients with no evident AML appearance. We observed that both P2X7A and P2X7B mRNA levels were strongly increased in *de novo* AML as compared to MDS, supporting the hypothesis that both receptor variants positively correlate with disease progression [42]. Interestingly, AML relapsing patients presented with a pathology return after the first therapeutic intervention were characterized by the differential expression of P2X7A and P2X7B compared to *de novo* patients. Indeed, while P2X7A expression was significantly reduced, P2X7B mRNA remarkably increased, suggesting that chemotherapy may cause the death of P2X7A expressed leukemic blasts and, conversely, did not affect P2X7B expressed leukemic blasts in subjects unresponsive to the treatment [42]. On the contrary, in remitting AML patients, both P2X7A and B expression was significantly decreased. All patients experiencing a relapse in AML were treated with daunorubicin, which is a chemotherapeutic usually used in leukemia therapy [88] together or alternatively to citarabine and idarubicin. Interestingly, among these drugs, daunorubicin is the only one that causes a significant increase in extracellular ATP in the tumor microenvironment [64]. Therefore, we hypothesized that P2X7A expressed by AML cells in the presence of high ATP concentration triggers cell death via a large pore opening. At the same time, P2X7B, which is unable to form the cytotoxic pore, induces leukemic cell proliferation, facilitating disease relapse. To confirm our hypothesis, we reproduced the patients’ data in an in vivo model of leukemia using the HL60 human cell line expressing both P2X7 isoforms. Tumor-bearing mice were treated with daunorubicin and with a P2X7 antagonist, alone or in co-administration. Both compounds significantly reduced leukemia growth, but their co-administration was more efficacious than single-drug treatment [42]. Daunorubin’s effect on P2X7 isoforms expression was similar to patients’ data, as P2X7B expression was significantly increased by daunorubicin, while P2X7A showed a tendency to decrease. To study the effect of daunorubicin on P2X7 isoforms, we took advantage of HEK 293 transfected with P2X7A and P2X7B separately [8]. Interestingly, daunorubicin toxicity was significantly increased in HEK P2X7A compared to controls, while the expression of P2X7B protected the cells from daunorubicin-dependent death. Thus, we wondered whether P2X7A-mediated pore opening could facilitate the entrance of daunorubicin into tumor cells, as it was demonstrated in macrophages for its analogous doxorubicin [89]. Thanks to HEK P2X7A and HEK P2X7B, we demonstrated that this phenomenon was solely dependent on P2X7A, which is the only isoform that significantly increases daunorubicin uptake. These data led us to hypothesize that a combined therapy comprising as a first step daunorubicin administration, to eliminate leukemic cell expressing P2X7A, and as second step P2X7B blockade, on blasts resistant to the toxicity effect of daunorubicin, will be a suitable approach in AML treatment [42]. Moreover, our data suggest that while, on the one hand, the overexpression of P2X7A will cause an initial proliferative advantage to myoblasts favoring their leukemic transformation, once chemotherapy is administered, its cytotoxic/pore forming activity will favor disease regression. On the other hand, P2X7B overexpression will only confer trophic/transforming charachteristics to myeloblast and even offer an advantage to leukemic blasts in the resistance to chemotheraphy.

P2X7J variant was initially isolated from cervical cancer cells as a 1652 bp mRNA. This alternative transcript gives rise to a protein of 258 amino acids, lacking part of the extracellular domain plus the entire second transmembrane domain and the C terminal tail and acquiring ten altered unique residues in its new carboxyl-terminal domain (Figure 1 and Figure 2) [35]. P2X7J acts as a dominant-negative isoform on P2X7A by reducing its cytotoxic activity and seems to be over-expressed in cervical cancer cells compared to the healthy cervical counterpart [35] (Table 1). Unfortunately, early studies on P2X7J published around 15 years ago have not been followed by further reports analyzing the role played by this isoform in other oncologic diseases. Interestingly, P2X7J expression was also retrieved in the cell nuclear fraction [35], suggesting that this variant might even play a role in regulating the expression of other P2X7 variants or related genes as lately hypothesized for P2X7-V3 [43].

nfP2X7 was the object of numerous tumor-related studies due to its widespread expression in cancer specimens [45,90,91,92] and to the efficacy of antibodies targeting it in basal cell carcinoma [83] (Table 1). This conformational variant of the P2X7 receptor seems to be more expressed than P2X7A on tumor cells and biopsies, promoting cell viability [45]. These features are highly reminiscent of P2X7B, which was demonstrated to be overexpressed compared to P2X7A in both solid cancer and leukemia, where it increased survival in serum starvation and even protected cells from chemotherapy-dependent cell death [37,42]. Moreover, high ATP concentrations, comparable to those present in the tumor microenvironment, promote cell surface expression of nfP2X7 while reducing P2X7A membrane localization and macropore-forming activity [45]. Finally, the same authors showed that in tumor specimens, nfP2X7 could also be localized at a subpopulation of B-lymphocytes and monocytes, albeit it was mainly concentrated on cancer cells [45]. The study by Gilbert and colleagues gave essential insights on the nature of nfP2X7 and was central in reinforcing the experimental and clinical data suggesting an efficacy as an anti-tumoral drug of BIL03s, which is a polyclonal antibody specifically targeting nfP2X7. Nevertheless, the possibility of analyzing this protein’s primary structure will significantly advance the comprehension of the mechanisms leading to these therapeutical benefits. Moreover, BIL03 was so far administered only as an ointment on the epidermis of patients or felines affected by basal cell carcinoma [83,93]. The testing of its efficacy as a systemic agent will open the way to extend it as a possible therapeutic option to cure a higher number of oncologic conditions.

Finally, an intriguing splice variant of human P2X7 is P2X7-V3, which was attributed a role as long non-coding RNA promoting uveal melanoma transformation [43] (Table 1). Pan and colleagues identified this transcription variant in two uveal melanoma cell lines and demonstrated that its silencing reduced tumor burden in a murine model. Interestingly, the main pathways negatively affected by P2X7-V3 silencing were the same activated by P2X7 functional protein. Indeed, the authors showed by immunohistochemistry that in tumors lacking P2X7-V3, there was a significant reduction of the proliferation marker Ki-67, which was also upregulated in tumoral models by P2X7A expression [70]. Similarly, Cadherin and Vimentin are positively regulated by P2X7A and B [40] and can be down-modulated by P2X7-V3 silencing [43]. Moreover, by genome-wide analysis, Pan and colleagues identified P2X7-V3 as a potent enhancer of the PI3K/AKT pathway that is, again, well known to be activated downstream P2X7 receptor in cancer [72,77,94,95]. All these data suggest that P2X7-V3 could act as a direct regulator of the receptor’s functional splice variants. Nevertheless, the aforementioned study is centered on the silencing of the variant. Still, it does not show whether the construct used in silencing experiments, although designed to target P2X7-V3 specifically, can down-modulate other P2X7 splice variants.

### 2.3. P2X7 SNPs in Oncology

A defined number of studies also analyzed the association between P2X7 SNPs and cancer occurrence or prognosis (Table 2). Most of these studies reported only genetic associations between the P2X7 SNPs and tumoral progression or related symptoms to identify new biomarkers for the analyzed diseases without dealing with an in-depth analysis of receptor function. The most studied P2X7 polymorphic variant was the 1513 A > C (E496A), which, as mentioned above, causes a loss of function of the receptor. A study conducted on B-chronic lymphocytes leukemia (B-CLL) showed a correlation between this SNP, both in heterozygous and homozygous forms, and familial B-CLL occurrence [96]. Moreover, cells carrying the mutated allele when treated with ATP at high concentration died less than those expressing the wild-type receptor. Based on these data, the authors inferred that leukemic cells expressing 1513 A > C could escape apoptosis, which is a feature generally associated with a bad prognosis in B-CLL [96]. At the time of the publication of the study mentioned above, the trophic function of P2X7 was far from being established, while the common view on the scientific community was that P2X7 acted mainly as a cytotoxic pro-apoptotic receptor [97]. Therefore, it is not completely surprising that a following study on 1513 A > C in B-CLL reported opposite results [98]. Indeed, Thunberg et al. reported a better survival outcome for 1513 A > C CLL patients than those with the wild-type allele. The authors explained these contrasting data, suggesting that this P2X7 SNP could induce different effects according to disease phases. It could promote tumor development in the early stages, while in the advanced phases, it could be protective [98]. These two preliminary studies [96,98], together with another report associating P2X7 overexpression with B-CLL progression [99], lead to the analysis of the 1513 A > C polymorphism in populations of different geographical origin, which failed to prove any positive or negative association of the SNP with B-CLL progression [100,101]. These discording data could depend upon other factors such as the contemporary presence of other P2X7 SNPs, causing gain of function, which were not known at that time, or the expression of splice variants not including the amino acid undergoing the mutation. Although data on CLL were not encouraging, the search of 1513A > C as an oncogenic marker was pursued in studies centered on other malignancies (Table 2). In a study carried out on patients affected by papillary thyroid cancer, 1513A > C SNP expression was not significantly different compared to healthy control. However, when patients were subdivided according to the histological subtypes in follicular variant and classical papillary carcinoma, the frequency of the minor allele was found higher in the follicular variant. Moreover, this polymorphism was found positively correlated with tumor dimension and with a high grade of malignancy, suggesting that 1513C could be a potential negative prognostic factor [102] (Table 2). This phenotype could be linked to the role played by P2X7 receptor in the immune respose against cancer also during immunogenic cell death (ICD). ICD is a particular type of cell death caused by some chemothereputics such as anthracyclines and characterized by the activation of a specific immune anticancer response that determines a favorable long-term outcome. During ICD, some molecules, such as ATP, are released from tumor dying cells as danger signals and activate, through the P2X7 receptor, the inflammasome in macrophages, inducing the maturation and the release of IL-1β that promotes tumor antigens presentation to dendritic cells. In breast cancer patients treated with antracyclines, the 1513 C genotype reduces the affinity for ATP, decreasing the release of IL-1β and in consequence the activation of the immune response during ICD [103]. In multiple myeloma, the frequency of 1513 C was not different between patients and healthy control nor was it associated with age at onset, level of beta-2 microglobulin, amount of hemoglobin and creatinine, time of response to therapy, and overall survival rate [104] (Table 2). Finally, the 1513 C SNP, when coexpressed with a loss of function SNP of the VEGF receptor, is associated with lower aggressiveness of disease [105]. More recent studies performed the analysis of multiple P2X7 SNPs in oncological conditions. For example, a case-control study performed on a Chinese population investigated 1513A > C, 946G > A, 1096C > G, and 1068G > A polymorphisms in association with hepatocellular carcinoma susceptibility [106]. This genetic analysis suggested no association between 1096C > G SNP and hepatocellular carcinoma. On the contrary, 1513A > C and 946G > A were correlated to a high risk to develop the disease, while 1068G > A was associated with a low risk to hepatocellular carcinoma onset (Table 2). This was the first study suggesting a role in tumor development for both the 946G > A and 1068G > A variants; therefore, further insight is needed to fully understand their role in hepatocellular carcinoma development and their potential as therapeutic targets. Finally, in an investigation conducted on a cohort of women with breast cancer who developed chronic pain after mastectomy, the gain of function 489C > T SNP was associated with high-intensity pain sensation, while the loss of function 835G > A was associated with a decrease of suffering [58] (Table 2). These data suggest that P2X7 genotyping could be useful to find a personalized strategy to relieve pain in terminal oncologic patients [58].

## 3. Conclusions

An increasing body of literature correlating P2X7 variants with tumoral insurgence and progression has emerged in recent years, testifying to the central role of these proteins and mRNAs in oncology. Recent evidence that appeared on P2X7B and nfP2X7 isoforms strongly suggest that variants lacking the pore-forming cytotoxic activity are central in oncologic progression and response to current anti-tumoral therapies and offer fascinating insights for the development of new therapeutic strategies. Although further efforts are required to understand better the interplay among P2X7 variants in cancer evolution and in the tumor–host interplay, we firmly believe that this investigation field will be fertile in the years to come and pave the way for the testing of P2X7 targeting drugs in oncology.

## Figures and Tables

**Table 1:** Studies reporting an association among P2X7 splice and conformational variants and cancer.

P2X7 Variants	Type of Cancer	Effects/Correlation
P2X7B	Osteosarcoma [37,39]	Cancer promotion
	Neuroblastoma [38]	Metastasis promotion
	Glioblastoma [40]	Invasion promotion
	Lung adenocarcinoma [41]	Positive correlation
	Acute myeloid leukemia [42]	Cancer progression
P2X7J	Cervical cancer [35]	Positive correlation
nfP2X7	Basal cell carcinoma [45,83]	Increase cell viability
P2X7-V3	Uveal melanoma [43]	Cancer promotion

**Table 2:** Studies reporting an association among P2X7 polymorphisms and cancer.

SNPs/aa Changes/dbSNPID	Type of Cancer	Effects/Correlation
1513A > C/E496A/rs3751143	B-chronic lymphocytes leukemia [96,98,100,101]	Cancer promotion/protection
	Papillary thyroid cancer [102]	Correlation with a high grade of malignancy
	Breast cancer [103]	Decrease of the anti-tumoral immune response
	Multiple myeloma [104]	No correlation
	Prostate cancer [105]	Correlation with low aggressiveness
	Hepatocellular carcinoma [106]	Positive correlation
835G > A/H270R/rs7958311	Breast cancer [58]	Low pain sensation
946G > A/R307Q/rs28360457	Hepatocellular carcinoma [106]	Correlation with a high risk of cancer development
1096C > G/T357S/rs2230911	Hepatocellular carcinoma [106]	No correlation
1068G > A/A348T/rs17118119	Hepatocellular carcinoma [106]	Correlation with a low risk of cancer development
489C > T/H155Y/rs208294	Breast cancer [58]	High pain sensation
